# In Silico Analysis to Explore Lineage-Independent and -Dependent Transcriptional Programs Associated with the Process of Endothelial and Neural Differentiation of Human Induced Pluripotent Stem Cells

**DOI:** 10.3390/jcm10184161

**Published:** 2021-09-15

**Authors:** Maryam Nakhaei-Nejad, Luke Trinity, Hosna Jabbari, Manijeh Pasdar, Nadia Jahroudi

**Affiliations:** 1 Department of Medicine, University of Alberta, Edmonton, AB T6G 2S2, Canada; maryam@ualberta.ca; 2 Department of Computer Science, University of Victoria, Victoria, BC V8P 5C2, Canada; ltrinity@uvic.ca (L.T.); jabbari@uvic.ca (H.J.); 3 Department of Oncology, University of Alberta, Edmonton, AB T6G 2H7, Canada

**Keywords:** induced pluripotent stem cells, differentiation, endothelial cells, neural cells, transcription factors, epigenetic regulators

## Abstract

Despite a major interest in understanding how the endothelial cell phenotype is established, the underlying molecular basis of this process is not yet fully understood. We have previously reported the generation of induced pluripotent stem cells (iPS) from human umbilical vein endothelial cells and differentiation of the resulting HiPS back to endothelial cells (Ec-Diff), as well as neural (Nn-Diff) cell lineage that contained both neurons and astrocytes. Furthermore, the identities of these cell lineages were established by gene array analysis. Here, we explored the same arrays to gain insight into the gene alteration processes that accompany the establishment of endothelial vs. non-endothelial neural cell phenotypes. We compared the expression of genes that code for transcription factors and epigenetic regulators when HiPS is differentiated into these endothelial and non-endothelial lineages. Our in silico analyses have identified cohorts of genes that are similarly up- or downregulated in both lineages, as well as those that exhibit lineage-specific alterations. Based on these results, we propose that genes that are similarly altered in both lineages participate in priming the stem cell for differentiation in a lineage-independent manner, whereas those that are differentially altered in endothelial compared to neural cells participate in a lineage-specific differentiation process. Specific GATA family members and their cofactors and epigenetic regulators (DNMT3B, PRDM14, HELLS) with a major role in regulating DNA methylation were among participants in priming HiPS for lineage-independent differentiation. In addition, we identified distinct cohorts of transcription factors and epigenetic regulators whose alterations correlated specifically with the establishment of endothelial vs. non-endothelial neural lineages.

## 1. Introduction

Cellular differentiation is fundamental in the development of multicellular organisms and has long been a subject of intense investigation. With current advancements in gene and cell therapy, a clear understanding of the cellular processes that are evoked to establish a specific cell phenotype are central towards designing appropriate cellular manipulations for therapeutic purposes. Among various cell types, endothelial and neural cells are highly attractive targets for therapeutic manipulation, due to their involvement in a multitude of prevalent vascular and nervous system disorders. Dysfunction of endothelial cells that cover the lumen of all blood vessels is a major contributing factor to many cardiovascular, peripheral vascular and cerebrovascular diseases, and many diseases of the central nervous system involve damage to neurons/neural cells. Exploration of the mechanism of endothelial cell specific gene regulation, as well as gene knock out animal models and stem cell differentiation approaches have led to identification of a number of transcriptional regulators that have emerged as major participants in the establishment of the endothelial cell phenotype [1,2,3,4]. These include: members of GATA, forkhead (FOX), Kruppel-like factor (KLF), SRY-box containing (SOX) and E-26-specific (ETS) transcription factor families [2,3,5], among which ETV2 (a member of the ETS transcription factor family) is a highly prominent contributor to the establishment of the endothelial cell phenotype [6]. Similar approaches have led to identification of a number of neural lineage specification factors, such as distinct members of the basic Helix Loop Helix (bHLH) family of transcription factors, including NGN family members, ASCL1, OLIG2 and NEUROD, as well as non-bHLH factors, such as PAX6 and SOX2 [7,8]. Nevertheless, despite major advancements in molecular and cellular biology, notable gaps remain in our knowledge regarding the molecular basis of cellular differentiation. While some master regulators of cell fate determination have been identified for some lineages, such as myogenic transcription factor MyoD, they still remain unknown for many others, including endothelial cells [9,10]. Specifically for endothelial cells, evidence pointing to a combinatorial role for ETS and FOX in regulating a number of endothelial specific genes, has led to an emerging consensus that establishment of endothelial cell phenotype requires more than a single master regulator [11]. This notion of a combinatorial role for transcriptional regulators has been extended to those that participate in neural cell fate determination, based on a recent CRISPR gene activation screening approach [12]. Thus, although the focus of intense investigation, the mechanisms of establishment of endothelial and neural cell lineages are still not fully understood [5,6,12,13,14]. Additionally, it is not yet understood how differentiation towards one lineage is selected over another, and what are the common vs. distinct processes that contribute to cell fate determination.

The generation and manipulation of stem cells, especially induced pluripotent stem (iPS) cells, combined with high throughput gene expression and computational biology advancements, have provided unprecedented opportunities to design and implement in vitro experimental approaches towards addressing these questions. These approaches have been successfully used to generate a wealth of information regarding changes in gene expression patterns that accompany the establishment of specific cell phenotypes, including endothelial and neural cells [15,16]. However, they generally involve either the comparison of terminally differentiated cells with each other, or a comparison of a specific lineage, which the stem cell used to derive the target differentiated cell phenotype. While the information provided from these studies leads the way towards identification of potential regulatory networks that are necessary for the establishment of a target cell phenotype, they do not allow unambiguous distinction between networks that are activated to establish a unique phenotype from those that may be activated for the differentiation process regardless of the ultimate phenotypic destination.

A potential approach towards addressing this gap in knowledge is to compare the molecular signature of two distinct cell lineages that are derived from one parental stem cell, thus possessing a similar genetic background with each other as well as with the parental stem cell. We have previously reported the generation of induced pluripotent stem cells (iPS) from human umbilical vein endothelial cells (HUVEC) the and subsequent differentiation of the resulting iPS (referred to as HiPS) back into a highly pure population of endothelial cells (Ec-Diff), as well as a population of neural cells that contained both neurons and astrocytes (Nn-Diff) [17,18]. Although the Nn-Diff were a mix of both neurons and astrocytes, their common neural phenotype is clearly distinct from endothelial cells, and thus they provide an appropriate comparator to the highly pure endothelial population of Ec-Diff.

A comparison of HUVEC, HiPS and EC-Diff transcriptome by gene array provided information regarding transcription regulatory factors, including chromatin and DNA modifiers, as well as cofactors as potential contributors to the establishment of endothelial cell phenotype [18]. Furthermore, we had performed gene array analyses of Nn-Diff that were derived from HiPS to confirm their neural lineage identity [17]. Here, using the two datasets generated from these two analyses [17,18], we have identified genes that are specifically associated with the establishment of endothelial compared to neural phenotypes. We aimed to gain insight into transcriptional machinery that is activated commonly to allow a general differentiation process compared to those that are associated with differentiation specifically towards endothelial or non-endothelial neural lineage.

## 2. Results and Discussion

### 2.1. Grouping of Genes That Exhibit Significant Differential Expression

**Figure 1:**
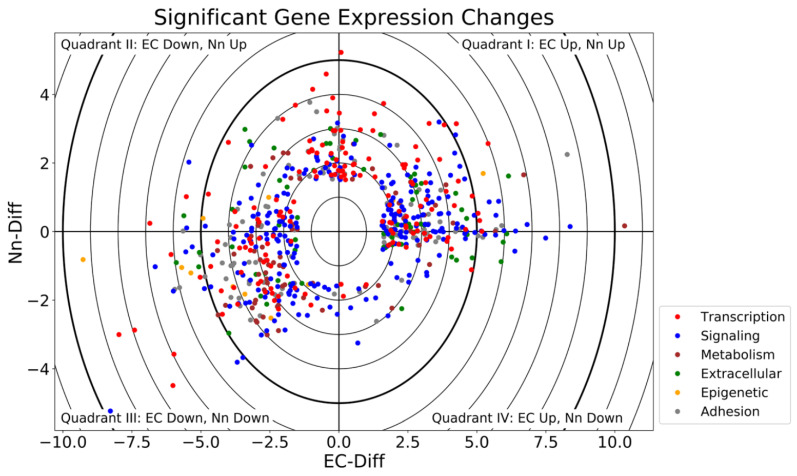
**Schematic representation of the differentially expressed genes.** Each quadrant represents genes that were positively or negatively altered when HiPS were differentiated towards neural (Nn-Diff) or endothelial (EC-Diff) cell phenotypes. Quadrant I (top right) upregulated in EC-Diff and Nn-Diff. Quadrant II (top left) downregulated in EC-Diff, upregulated in Nn-Diff. Quadrant III (bottom left) downregulated in EC-Diff and Nn-Diff. Quadrant IV (bottom right) upregulated in EC-Diff, downregulated in Nn-Diff. Colors correspond with the gene’s functional classification.

Based on the criteria described in Methods, genes that were significantly altered in HiPS-derived endothelial (EC-Diff) or neural (Nn-Diff) cells (relative to HiPS) were identified and a scatterplot of differentially expressed genes generated. Figure 1 demonstrates a total of 644 differentially expressed genes (increased or decreased).

To gain insight into the nature of the genes that were altered, they were sorted according to gene ontology, generating a hierarchical structure that is based on their functional characteristics [19]. Genes were sorted based on six gene ontology classifications, which were expected to be relevant in biological processes associated with differentiation. These included transcription factors, epigenetics, signaling, cell adhesion, extracellular matrix and metabolism.

**Figure 2:**
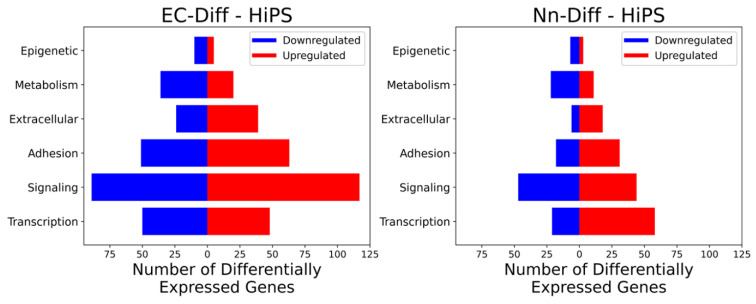
**Subplots of differentially expressed genes based on their ontology**. The subplots show genes that were differentially expressed in endothelial (Ec-Diff-HiPS) or neural (Nn-Diff-HiPS) lineages compared to HiPS, both upregulated (red) and downregulated (blue). In both subplots, the genes are stratified into the six functional ontologies, indicated on the *y*-axis. The *x*-axis shows the number of genes that were up- or downregulated in each lineage.

For both endothelial and neural lineages, signaling was the largest group of genes with significant changes, followed by transcription factors and adhesion subsets (Figure 2). The lowest number of genes that displayed significant changes belonged to the epigenetic ontology group. Considering the extended network of genes that are targets of any one epigenetic regulator, it is not surprising that alterations in the expression of a small number of epigenetic regulators may have an amplified effect on the target cell gene expression profile. Target genes of epigenetic regulators include transcription factors, which in turn regulate the expression of multiple target genes of their activity. The differentially altered genes in endothelial and neural cells relative to iPS were classified into six categories. The categories included genes that were (i) upregulated in both, (ii) downregulated in both, (iii, iv) up- or downregulated in one but unchanged in the other and (v, vi) upregulated in one and downregulated in the other. This report is focused on ontology classes of transcription factors (Tables 1–7) and epigenetics (Tables 8–12) since the regulation of gene expression is the major determinant of establishing cell phenotype.

### 2.2. Characterization of Transcription Factors That Participate in a General Differentiation Process

We propose that transcription factors that are upregulated in both lineages (Table 1) participate positively in the initiation of the differentiation process towards a multipotent progenitor. Nine genes were identified in this category, which included two members of GATA transacting factors (GATA3 and GATA6) in addition to ZFPM2 (friend of GATA family member 2, which acts as a coactivator or corepressor of GATA family members [20,21]), RARB [22], NR2F2 (also known as COUPTFII, with a role in venous endothelial specification, and neural crest cell development [23]), HOXB2 (involved in neurogenesis [24]), MEF2C (involved in neuronal and endothelial development [25]), HOXA4 (involved in stem cell differentiation but not specific to endothelial or neuronal [26]) and ZNF436 [27]. The majority of these genes were upregulated in both lineages to comparable levels (either equal or at most 2–3-fold differences), except for GATA6, which was upregulated at a significantly higher level in endothelial compared to neural cells (an approximately 7-fold difference).

Furthermore, we suggest that transcription factors, which were downregulated in both lineages (Table 2), are inhibitory towards both differentiation pathways. Thirteen genes were identified in this category. They included factors that have been reported as necessary to maintain an undifferentiated stem cell state, such as NANOG, POU5F1(Oct-4), NR5A2 (LRH-1) [28], those recognized as stem cell marker including ZFP42/Rex1 (also reported to have epigenetic regulatory function [29]) and FOXD3 (a repressor with epigenetic function involved in the transition from naïve to primed pluripotent stem cells, but also acts as a repressor of maximal activation of target promoters [30]). This category also included factors that predominantly participate in regulating differentiation towards endothelial and/or neuronal lineages. For instance, SOX8 is reported to play a role in embryonic development, but also participates in neuronal cell fate determination in a transient and time-dependent manner [31]. Additionally, transcription factors in this category included those with a role in non-neuronal/endothelial cell phenotypes, for example FOXA3 with a role in establishing hepatic cell fate and ETV1, which is reported to participate in the establishment of His–Purkinje system development [32,33]. This category also included genes for factors that function as repressors of other transcription factors, or partner with some factors that are required for maintaining pluripotency. For instance, HEY2 functions as a repressor of GATA6, as well as partnering with histone decaytalases (HDACs) and promoting its epigenetics transcriptional repressive function [34,35]. ZNF165 partners with SMAD3, and FOXH1 partners with SMAD2 [36,37]. Both SMADS 2 and 3 (transcriptional targets of the TGFb signaling pathway) are reported to play a role in the maintenance of pluripotency [38]. It is noteworthy that two pseudo genes POU5F1P3 and NANOGP1 were also significantly downregulated, suggesting a potential functional relevance of the expression of these genes. Based on its homology to NANOG and its high transcripts levels in stem cells, it is conceivable that NANOGP1 may participate in the maintenance of stem cells phenotype [39]. POU5F1P3 expression was reported to produce a cytoplasmic protein in undifferentiated cells (as well as some cancer cells) that is downregulated during neuronal differentiation [40,41].

The coregulation of GATA 3 and 6 transacting factors, together with their regulators ZFPM2 (FOG2) and HEY2, may suggest their similar contribution towards differentiation of both neuronal and endothelial lineages. Among the genes upregulated in both lineages (NR2F2 (COUPTFII), MEF2C, HOXB2, HOXA4 and RARB), NR2F2 (COUPTFII) and MEF2C have previously identified roles in both endothelial and neuronal lineage specification [23,25]. HOXB2, HOXA4 and RARB present as novel candidates that may be involved commonly in specification of both lineages.

### 2.3. Transcription Factors That Participate in Establishment of Endothelial Cell Phenotype

#### 2.3.1. Transcription Factors That Are Upregulated in Endothelial Cell Lineage

We propose that transcription factors that are specifically upregulated in endothelial cells but remained unchanged in neuronal cells are those that actively participate in establishing endothelial cell phenotype (Table 3). The list of 39 transcription factors identified in this category included previously identified endothelial-enriched transcription factors, such as ETS1, TAL1, ERG, FLI1, FOXC1, as well as others, such as NFIB, that are not reported to be specifically restricted to endothelial lineage [2,42]. The GATA2 member of the GATA family of transcription factors was also among those that were specifically upregulated in the endothelial lineage. Thus, it appears that the GATA family of transcription factors plays important roles in differentiation processes, and while GATA3 and 6 may contribute to the multilineage progenitor state during differentiation, GATA2 is specifically activated during endothelial cell commitment.

Notably, the top 10 most highly upregulated transcription factors in this group were ERG, FLI1, TAL1, EPAS, MECOM, TFEC, HOPX, SOX17, SOX7 and SOX18. Among this group, ERG and FLI1 are the members of the ETS family of transcription factors with well-established endothelial-specific gene regulatory function [43]. TAL1 has long been recognized as an endothelial-specific transcription factor that is required for embryonic vascular remodeling and angiogenesis, while not being detected in quiescent endothelial cells [44]. Thus, its upregulation in cultured HiPS-derived endothelial cells may represent the proliferative as well as endothelial phenotypic state of these cells. EPAS1 (also known as HIF2A) is an endothelial-specific hypoxia-responsive transcription factor, which has been proposed to participate in the formation of blood vessels, specifically in response to low oxygen levels [45]. Thus, its high expression level here may suggest that oxygen level response could be a major initiating event that primes progenitor cell differentiation towards a mature endothelial cell phenotype. Hence, a high level of this transcription factor may establish the cellular physiology for such response.

MECOM is a transcription factor that, in addition to DNA-binding activity, exhibits chromatin-modifying functions [46,47]. It interacts with repressor CtBP and histone-modifying coactivator PCAF (KAT2B), with histone acetylase activity [47,48]. MECOM itself also has histone methyl transferase activity [49]. TFEC is another transcription factor, which was not previously reported to have a known role in endothelial-specific gene regulation. However, it is emerging as a participant in promoting the vascular bed-specific function of distinct endothelial cells [50,51]. TFEC supports the hematopoietic stem cell (HSC) expansion function of endothelial cells in the HSC vascular niche and regulates the expression of cytokines by these endothelial cells [51]. HOXP is among the identified transcription factors and was previously shown to be required for optimal primitive hematopoiesis and hemato-endothelial progenitor differentiation [52]. While lacking DNA binding activity, HOXP exhibits cofactor function, as well as epigenetic regulation through the recruitment of histone modifying factors, such as histone deacetylase (HDAC), and consequently influencing the chromatin modification of target genes [53].

The SOX family of transcription factors is known to participate in cell fate determination, including maintenance of stem cell phenotype [54]. Indeed, SOX2 was among the six transcription factors that we used to generate the original HiPS from endothelial cells [17]. There are more than 20 SOX family members, which are divided into eight groups (A-H) based on their DNA binding domain amino acid sequence [55]. SOX7, SOX17 and SOX18 identified here are members of the SOX F family that are upregulated in endothelial cells [56]. Interestingly, while SOX family members were not identified among genes that were upregulated commonly in both endothelial and neuronal lineages (Table 1), SOX8, a member of the E group, was downregulated in both lineages (Table 2). Additionally, as will be discussed below, there were no SOX family members identified among transcription factors that were specifically up- or downregulated in the neuronal lineage. However, as discussed below, specific SOX family members were downregulated in endothelial cells. These data present the SOX family of transcription factors (particularly F family members) as a major participant in specifically establishing the endothelial cell phenotype.

#### 2.3.2. Transcription Factors That Are Downregulated in Endothelial Cell Lineage

The establishment of endothelial cell phenotype may also be associated with the repression of specific transcription factors, which if present would interfere with the process. While low levels of some transcription factors may indicate no significant change during endothelial differentiation, others may be actively repressed, which could suggest a requirement for their specific downregulation to promote endothelial differentiation. Analyses of transacting factors that are specifically downregulated in endothelial cells (Table 4) will provide a window towards identifying potential factors in the latter category. Various members of the SOX, ZIC and SALL family of transcription factors, as well as OTX2 and ZNF90 are the top 10 downregulated transcription factors in this category. SOX2, SOX11 and SOX21 and ZIC2, ZIC3 and ZIC5 are multifunctional transcriptional regulators with DNA and protein binding domains that interact with various transcription and chromatin-modifying factors [57]. SALL1 and II are known as transcriptional repressors that contain multiple domains, which interact with the histone deacetylase complex or heterochromatin [58]. The other two transcription factors among the top 10 downregulated genes are OTX2, which participates in early neuronal, as well as brain and sensory organ development [59], and ZNF90, which is a protein with proposed DNA binding and as yet undefined transcriptional regulatory function based on information for the ZNF90 Gene in GeneCards (https://www.genecards.org/cgi-bin/carddisp.pl?gene=ZNF90, accessed on 23 April 2021).

The results presented here suggest the establishment of endothelial cell phenotype as being associated with the upregulation of specific SOX, ETS and GATA transcription factor family members, TFEC and HOXP that participate in hemato-endothelial progenitor establishment, EPAS1 that is responsive to oxygen levels and transcription factor MECOM with multiple chromatin modification functions. It is noteworthy that a number of these transcription factors have an expression pattern that is not restricted to endothelial cells, suggesting a potential combinatorial role for this cohort of transacting factors in establishing endothelial cell phenotype. Additionally, of interest is the GATA transcription factor family, which includes members such as GATA2 that participate in endothelial lineage establishment, and others such as GATA3 and 6 that are associated with the lineage-independent differentiation process.

Among the factors that were downregulated in endothelial cells, two were of special interest since they were specifically upregulated in neuronal cells (Table 5), thus suggesting that these factors may perform direct opposing functions in the establishment of endothelial vs. neuronal phenotype. These were IRX2 and IRX3, which belong to the Iroquois homeodomain transcription factor family [60] and will be discussed in more detail later.

### 2.4. Transcription Factors That Participate in Establishment of Neural Cell Phenotype

#### 2.4.1. Transcription Factors That Are Upregulated in Neural Cell Lineage

Based on similar reasoning to that used for endothelial cells, we propose that genes that are specifically upregulated and downregulated in neural cells are those that participate in the establishment of neural cells phenotypes. In the category of the genes that were upregulated in neural cells (Table 6), the majority did not show a significant change in endothelial cells, while two genes were identified that were specifically downregulated in endothelial cells (Table 5). The top 10 most upregulated neural lineage-specific genes included ASCL1, POU3F2, ST18, TFAP2B, TFAP2A, PAX6, NR2E1, PITX2, HOXA1 and ARX (Table 6). A high proportion of these genes belong to homeobox-containing genes, and many have been previously shown to participate in the establishment of the central nervous system. ASCL1 (a member of the basic helix-loop-helix family) is a prominent pro-neuronal pioneering factor that, when appropriate chromatin modification signature is present, can bind nucleosomal DNA as a single factor [61]. ASCL1 and POU3F2 (homeobox family) (in combination with MYTL1, a member of the 3 zinc finger family of Myelin Transcription Factors (MYT)), were shown to be sufficient for the reprogramming of some non-neuronal somatic cells into neuronal lineage in vitro [62,63]. ST18 is also an MYT family member that is proposed to participate in neuronal differentiation [64,65]. MYT family members promote neurogenesis by functioning as repressors of non-neuronal genes through the recruitment of the histone deacetylase complex to target genes [66,67]. The two members of the TFAP family of transcription factors (TFAP2A and TFAP2B) were shown to activate gene sets that participate in the specification of neural crest cells. They participate in the regulation of gene expression by exchanging dimerization partners that subsequently recognize and read the epigenetic landscape of progenitor cells to promote neural crest specification [68]. Furthermore, TFAP2A and TFAP2B together with ASCL1, among the top 10 upregulated transcripts, exhibit an activation pattern that is highly dependent on the chromatin modification signature of their target genes [61,68,69], emphasizing the importance of epigenetics in establishing a neural-specific phenotype. Cortical development is highly dependent on Pax6, which is shown to function as an activator of neuronal lineage-specific genes, while repressing the expression of non-neuronal genes [70]. On the other hand, NR2E1 is necessary for neural stem cell self-renewal [71]. PITX2, HOXA1 and ARX are all homeobox-containing genes, which also participate in neuronal specification [72,73,74].

#### 2.4.2. Transcription Factors That Are Downregulated in Neural Cell Lineage

As discussed for endothelial cells, the establishment of neural lineage may also require downregulation of certain transcription factors. Seven transcription factors, including FOXO1, MEOX2, ZSCAN10, NFE2L3, HMX2, ARNTL2 and TCF19, were specifically downregulated in neural cells (Table 7). FOXO1, the most downregulated, is expressed in many cell types, including endothelial and neural stem/progenitor cells (NPC), while it is not detectable after transition from NPC to neuroblasts [75,76]. FOXO1 is recognized as a regulator of metabolic homeostasis and has been reported to mediate neuronal apoptosis in response to oxidative stress, growth factor deprivation, or depolarization [77,78]. FOXO1 null mice were shown to be embryonic lethal due to a defect in vascular development. Additionally, somatic deletion of FOXO1 in endothelial cells was shown to result in hyper proliferation. These observations indicate a role for FOXO1 in vascular homeostasis [75,79]. However, we did not detect FOXO1 among the genes that were specifically upregulated during the establishment of endothelial cell phenotype.

Another significantly downregulated transcription factor was MEOX2, which has a major role in vascular biology [80]. MEOX2 is a homeobox-containing protein that has been proposed to be a master negative regulator of angiogenesis in endothelial cells [81]. It also exhibits pro-apoptotic function, as well as an anti-proliferative effect on smooth muscle cells [80]. The decreased expression of FOXO1 and MEOX2 in neural lineage may be reflective of the requirement for downregulation of vascular development-related processes during neural differentiation. Furthermore, downregulation of these genes could promote neural cell fate establishment prior to the establishment of apoptotic programs that can be activated in response to various cues.

As expected, genes in this category not only included factors that are reported to participate in the maintenance of pluripotency, such as ZSCAN10 [82] and NFE2L3 [83], but also genes that participate in specialized neuronal differentiation as well as metabolic and circadian regulation. HMX2 participates in the specification of specialized neurons that express GHRH (growth hormone-releasing hormone), which control glucose metabolism and growth hormone secretion [84]. ARNTL2 is a regulator of genes that control circadian rhythms and immune cell proliferation [85,86]. TCF19 was shown to participate in the regulation of gluconeogenic genes and the maintenance of pancreatic beta cells [87,88]. It can also function as a transcriptional repressor by recruiting the chromatin modifier NuRD (nucleosome-remodeling deacetylase) complex to the target promoters [88].

Overall, these results suggest that differentiation towards neural phenotype is concomitant with the downregulation of genes that regulate: (i) specific cellular metabolisms, (ii) circadian cycles, (iii) response to external cues that could trigger apoptosis, (iv) vascular cell specification and (v) maintenance of stem cell phenotype.

### 2.5. Transcription Factors That Play Opposing Roles in Establishment of Endothelial and Neural Cell Phenotype

We also identified a number of transcription factors that were upregulated in one lineage and downregulated in the other. Identification of these factors provides a novel insight towards dual functioning transacting factors that may function as an activator of genes required for establishing one lineage while simultaneously repressing genes associated with establishment of a different lineage. We did not identify any transcription factors that were specifically upregulated in endothelial cells while being downregulated in neural cell lineage. However, we did identify two transcription factors, IRX2 and IRX3, that were upregulated in neural and significantly downregulated in endothelial cells (Table 5). IRX2 and IRX3 belong to the Iroquois homeobox family of transcription factors and were shown to participate in neurogenesis [60].

IRX2 was shown to participate in cerebellum formation and potentially the ventricular conduction system (VCS) [89,90], while IRX3 was shown to have a definitive role in establishing fast conduction in the VCS [90]. The downregulation of IRX2 and 3 in endothelial cell lineage suggested that these factors may have an inhibitory role in processes related to endothelial-specific function. However, IRX3 has been reported to participate in angiogenic activity of microvascular endothelial cells when stimulated with VEGF (vascular endothelial cell growth factor) [91]. Furthermore, IRX3 has been shown to have an endothelial expression pattern reflective of endothelial cells’ heterogeneity. More specifically, IRX3 is expressed in kidney endothelial cell in vivo [92]. IRX2, on the other hand, was shown to be present in tumor-associated endothelial cells but not normal endothelial cells [93,94]. The observation that IRX2 and 3 are upregulated in neural but downregulated in endothelial cells may suggest that these two factors are uniquely involved in cell fate determination by functioning as activators of neural cell fate-specific genes, while they are potentially repressors of endothelial cell fate-specific genes. The reported roles of these transacting factors in angiogenesis, and their tumor or kidney endothelial-specific association, suggest that the processes involved in the establishment of endothelial cell phenotype are distinct from those contributing to its further specification, such as the establishment of unique organ-specific characteristics, angiogenesis processes, or the development of specific attributes of a tumor vascular endothelial cell.

### 2.6. Epigenetic Regulators of Endothelial and Neural Cell Differentiation

Epigenetic modification of genes is another process that significantly contributes to activation/repression of gene expression [95]. As discussed above, some DNA binding transcription factors may also exhibit epigenetic functions either directly, or through association with chromatin-modifying factors (i.e., MEOM and ZIC), while others are known to function exclusively as epigenetic regulators. Considering the important role of epigenetics in regulating cell-type-specific gene expression, in our transcriptome analysis we identified a group of transcripts categorized as epigenetic regulators that were similarly or differentially expressed in endothelial and neuronal lineages (Tables 8–12).

#### 2.6.1. Characterization of Epigenetic Factors That Participate in General Differentiation Process

DNMT3B, PRDM14 and HELLS were identified as epigenetic regulators that were downregulated in both endothelial and neuronal lineages (Table 8). DNMT3B is a de novo methyl transferase enzyme that is shown to play a role in early stages of embryonic development [96]. DNA methylation is a well-established epigenetic modification that is regulated by DNA methyl transferases (DNMTs) and associated with transcriptional repression [97]. Thus, the downregulation of DNMT3B may contribute to hypomethylation, and consequently the priming of lineage-specific genes for transcriptional activation during the differentiation process.

PRDM14 (a member of the PRDM family of epigenetic regulators with histone methyl transferase activity [98]) is a DNA binding factor and epigenetic regulator that participates in the maintenance of pluripotency [99]. The epigenetic regulatory function of PRDM14 is correlated with its DNA demethylation, as well as histone methyl transferase activity [100]. Thus, the downregulation of PRDM14 in both neural and endothelial cell lineages suggests that it may specifically maintain pluripotency-related genes in a transcriptionally active chromatin structure. HELLS is a DNA helicase which also has a major role in regulating DNA methylation. Its chromatin remodeling activity is reported to render DNA accessible to DNMT3B, and it is also shown to interact with other epigenetic regulators, including DNMT1 (another member of DNMT family) and histone deacetylases HDAC1 and 2 [101,102]. Two of the three genes in this category are mainly involved in the regulation of DNA methylation, specifically that of de novo DNA methylation. Their downregulation may be necessary to prime the chromatin structure of differentiation-related genes for transcriptional activation. On the other hand, PRDM14, which is involved in DNA demethylation, appears to be recruited, through its sequence-specific DNA binding activity, to pluripotent-specific genes and maintain their transcriptional activity. Thus, PRDM14 downregulation may contribute to the repression of pluripotent-specific genes. Our analyses did not reveal direct epigenetic regulators that were upregulated in both endothelial and neural lineages. This demonstrated that the downregulation of factors that modify DNA methylation is a major and potentially primary epigenetic alteration that contributes to the repression of genes that participate in the maintenance of pluripotency and the activation of genes that initiate differentiation process.

#### 2.6.2. Epigenetic Factors That Participate in Establishment of Endothelial Cell Phenotype

Genes coding for epigenetic factors that were significantly altered in endothelial cells included three genes (PCGF5, LOXL2 and ERCC6) that were upregulated (Table 9) and eight genes (LIN28A, LIN28B, TET1, TRIM71, JARID2, PRKCB, CECR2 and H2AFY2) that were downregulated (Table 10).

PCGF5 is a histone modifier (mediates ubiquitylation of histone H2A) which exerts both repressive and activating functions and was reported to participate in neuronal differentiation [103]. Thus, its upregulation specifically in endothelial cells but not neural cells was unexpected. However, other reports demonstrated that PCGF5 participates specifically in mesodermal lineage differentiation and is associated with the expression of genes that regulate blood vessel morphogenesis [104]. This is consistent with our observed upregulation of PCGF5 in the mesodermally derived endothelial cells. LOXL2 is a lysyl oxidase, also a histone modifier. It alters histone H3 that is tri-methylated at lysine4 (H3K4me3); this is a signature of transcriptionally active chromatin [105,106]. It also targets deamination of other proteins, including transcription initiation factor subunits [107]. Thus, its epigenetic function is generally correlated to transcriptional repression, suggesting that its upregulation in endothelial lineage may promote the downregulation of non-endothelial-specific genes, including those necessary for maintenance of the stem cell phenotype. However, LOXL2 targets deamination of other non-transcription factor proteins, including collagen IV, which has been reported to promote angiogenic sprouting [108]. Thus, the specific LOXL2 upregulation in endothelial cell lineage may be representative of its role in angiogenesis independent of its epigenetic function. ERCC6 is a nucleotide excision repair factor that alters chromatin structure by wrapping DNA around itself to allow DNA damage repair at the site of active transcription [109]. Its epigenetic function stems from its chromatin remodeling activity which affects the binding of other transcription factors to their cognate binding site at the remodeled chromatin region [109,110]. Its upregulation specifically in endothelial cell lineage may indicate its yet-to-be-identified role in regulation of endothelial specific genes.

In addition to alteration in DNA methylation, histone modifications and chromatin remodeling, epigenetic regulation of gene expression may also encompass regulation of mRNA levels. This may involve the function of non-coding RNAs (such as miRNA and lncRNA) or RNA binding proteins that regulate target mRNA levels (including mRNAs coding for epigenetic regulators as well as transcription factors) [111,112].

Of the eight genes in the epigenetic category that were downregulated (Table 10), LIN28A, LIN28B and TRIM71 code for RNA binding proteins [113,114]. LIN28A and B are recognized as playing a significant role in maintaining pluripotency and exhibit an epigenetic function, mainly through recruitment of TET1, a DNA demethylation agent, to target genes [115]. In addition to its RNA binding and participation in the maintenance of pluripotency, TRIM71 was reported to have a role in early neural differentiation [116,117]. TRIM71 functions as a destabilizer or translational repressor of target mRNAs, including those whose downregulation is necessary for maintenance of the stem cell phenotype [114,118].

TET1 is a member of TET (ten–eleven translocation) family of dioxygenase enzymes that participate in DNA demethylation at the 5-methylcytosine (5mC) base, and thus contributes to an epigenetic modification generally consistent with transcriptional activation [119]. However, it also recruits transcriptional repressor complexes, including MBD3-NURD, with histone deacetylase and nucleosome remodeling activities, to specific target gene promoters, leading to transcriptional repression [119]. This repressive function of TET1 was proposed to be the dominating role in maintaining the inhibition of differentiation-related genes in stem cells [120]. JARID2 is a DNA binding protein that exerts an epigenetic regulatory function through association with and recruitment of histone methyl transferases, most notably the polycomb repressive complex 2 (PRC2) to target genes [121]. PRC2 functions as histone methyl transferase, leading to H3K27 methylation, a signature marker of a transcriptionally repressed gene [122]. However, JARID2 association with PRC2 may also exert an inhibitory effect, demonstrating JARID2′s dual function as an inhibitory or activating regulator of histone methyltransferase complexes, and consequently target gene repression or activation [123]. PRKCB is a member of the protein kinase C family, with a diverse role in various signaling pathways, and is known to phosphorylate a wide variety of proteins, including histone H3. PRKCB mediates the phosphorylation of Thr-6 on histone H3, a signature histone modification associated with transcriptionally active chromatin [124]. This epigenetic function of PRKCB is dependent on androgen signaling and occurs on promoters that are targets of androgen receptors [124]. CECR2 is a component of a chromatin remodeling complex and has been shown to participate in neurodevelopment [125,126]. Finally, H2AFY2 (macro H2A) is a core histone H2A variant found as a replacement for conventional H2A in a subset of nucleosomes, and consequently altering the target region chromatin structure in a manner that is associated with transcriptional repression [127,128].

#### 2.6.3. Epigenetic Factors That Participate in Establishment of Neuronal Cell Phenotype

The cohort of genes with epigenetic function that were altered in neural cells included two genes (BCORL1 and LHX2) that were upregulated (Table 11) and one gene (HENMT1) that was downregulated (Table 12). BCORL1 (BCL6 Corepressor Like 1) is a corepressor protein that is recruited to target genes through interaction with DNA binding proteins [129]. It also interacts with class II histone deacetylases and participates in the formation of the non-canonical polycomb repressive complex (PCR1.1) [129,130]. LHX2, known as the “cortical selector”, plays a central role in various processes of development of the central nervous system and neurogenesis [131]. LHX2 is a transcription factor that exhibits epigenetic function through interaction with nucleosome remodeling (NuRD) and histone deacetylase-containing complexes, and their recruitment to the target promoters [132]. The downregulated gene HENMT1 encodes for a methylating agent that targets the 3′ end of a short noncoding RNA, known as piRNA, for methylation [133]. This methylation step is a part of the maturation process of piRNA, which subsequently associates with an RNA cleaving complex containing PIWI/Argonaute and directs it to mRNA sequences targeted for cleavage [134].

Overall, epigenetic regulators, although generally lacking inherent specificity, exhibit differential expression patterns based on cellular phenotype and may exert lineage-specific effects based on their level of expression. Additional measures of specificity could be added through association of many epigenetic regulators with DNA binding transcription factors that recruit them to the target promoters. Recently, distinct RNA binding proteins have been identified as major epigenetic regulators, which exhibit lineage specificity and could present novel targets for exploring the epigenetic regulation of cell fate outside the chromatin structure [112,135,136].

## 3. Conclusions

**Figure 3:**
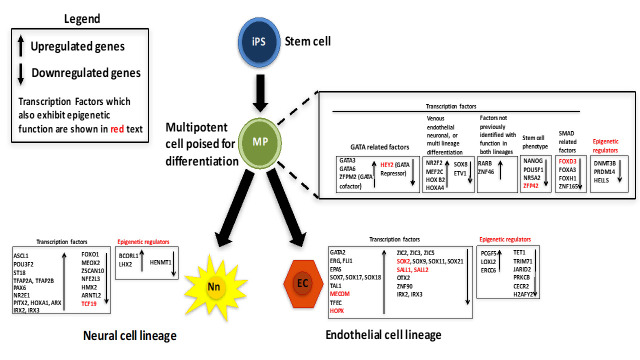
**Schematic representation of differentially expressed transcription factors/cofactors and epigenetic regulators when cells acquire the ability to differentiate, and when they are differentiated to EC vs. Nn lineages**. iPS, a cell that has stem cell phenotype; MP (multipotent), a cell that is poised to differentiate towards all or multiple lineages. Transcription factors that also exhibit epigenetic function are shown in red text.

Collectively, based on the results of the gene array analyses, we posit that the establishment of a distinct cell fate from pluripotency involves progression through stage(s) that independent of the final phenotype destination, poises the cell for differentiation. This process involves alterations in gene expression pattern compared to the pluripotency state and is independent of the final-destination phenotype. Once a cell is poised to differentiate, establishment of the final-destination phenotype (such as endothelial or neural cell fate) involves additional alterations in gene expression pattern that are lineage specific. Although these alterations include genes belonging to multiple functional groups, those involved in transcription regulation, including transcription factors and epigenetic regulators, are uniquely positioned to alter activities of multiple networks of target genes as master regulators. Thus, we focused on these two ontology groups and propose the following model (Figure 3) to describe the establishment of endothelial and neural cell lineages.

We hypothesized that transcription factor and epigenetic genes that are commonly up- or downregulated in both endothelial and neural lineages potentially promote lineage-independent pro-differentiation phenotypes. However, these analyses will not allow distinguishing among cells that may be poised to differentiation towards all, multiple, or a select few phenotypes. Furthermore, since cells differentiated into neural lineage constituted both neurons and astrocytes, the gene array analyses from these cells’ populations do not provide information regarding the neuronal- vs. astrocyte-specific gene expression profile. However, considering that both neurons and astrocytes belong to neural lineages, it remains informative with regard to exploring alteration in gene expression that occurs when iPS are differentiated towards endothelial compared to a distinct non-endothelial neural lineage. Notwithstanding the caveats, we propose that being poised to differentiate is associated with the downregulation of three epigenetic regulators (DNMT3B, PRDM14, HELLS) with major roles in regulating the DNA methylation profile. Since two of the three (DNMT3B and HELLS) are associated with de novo methylation, while PRDM14 has a demethylation function, their downregulation may alter the epigenetic landscape of a stem cell to prime lineage-specific genes for activation, while rendering stem cell-specific genes poised for repression. These observations highlight alterations in DNA methylation patterns as a major epigenetic event in priming a pluripotent cell for differentiation. Additionally, this process appears to be strongly associated with a major increase in GATA transcription factor activity, since an increase in the gene expression of select GATA family members (specifically GATA3 and 6) as well as GATA cofactor (ZFPM2), in combination with the downregulation of GATA repressor (HEY2), is prominent. The establishment of neural- or endothelial-specific lineages then requires additional gene expression alterations (in select epigenetic and transcriptional regulators) that are restricted to the target lineage. While these results require experimental confirmation, it provides a potential list of target genes that could be prioritized for experimental approaches to determine their role individually and/or collectively in differentiation process.

## 4. Materials and Methods

The initial dataset contained 49,400 observations for 19,929 unique genes [17,18]. Each observation had multiple gene expression values corresponding to induced pluripotent cells, differentiated endothelial cells and differentiated neuronal cells. Genes with significantly differential expression were selected using the fcros’ package in R in order to estimate the likelihood that a significant change in expression occurred [137,138]. This method detects differentially expressed genes based on the fold change rank, which is a ratio of means from control to test samples. The control expression used was gene expression from induced pluripotent cells. The two test expressions used were differentiated neuronal and differentiated endothelial cells, respectively. Genes were considered significantly upregulated if their adjusted *p*-value from the fcros’ package was above 0.95 and their mean change in expression was greater than 1.5. Similarly, genes were considered significantly downregulated if their adjusted *p*-value from fcros’ was below 0.05 and their mean change in expression was less than −1.5.

## Figures and Tables

**Table 1:** Genes in transcription factor category that are upregulated in both endothelial and neural cells.

	Gene	EC-Fold Change	Nn-Fold Change
1	GATA3	51.058	22.919
2	GATA6	42.389	5.923
3	ZFPM2	33.551	10.32
4	RARB	23.55	15.564
5	NR2F2	17.982	9.556
6	HOXB2	9.633	8.887
7	MEF2C	7.634	4.935
8	HOXA4	7.239	4.241
9	ZNF436	6.887	5.562

**Table 2:** Genes in transcription factor category that are downregulated in both endothelial and neural cells.

	Gene	EC-Fold Change	Nn-Fold Change
1	POU5F1	−334.824	−9.624
2	POU5F1P3	−169.194	−7.315
3	NANOG	−64.627	−22.43
4	ZFP42	−62.901	−11.91
5	NANOG///NANOGP1	−19.196	−3.314
6	FOXD3	−18.649	−5.317
7	NR5A2	−16.392	−13.733
8	SOX8	−13.993	−3.103
9	FOXA3	−12.691	−4.342
10	HEY2	−7.758	−4.113
11	ZNF165	−7.582	−2.87
12	ETV1	−6.688	−6.025
13	FOXH1	−6.352	−3.391

**Table 3:** Genes in transcription factor category that are upregulated in endothelial cells.

	Gene	Fold Change
1	EPAS1/// LOC100652809	117.891
2	SOX17	91.658
3	SOX7	74.419
4	MECOM	66.16
5	ERG	53.912
6	TFEC	50.453
7	FLI1	37.099
8	HOPX	36.998
9	SOX18	36.295
10	TAL1	34.351
11	ELK3	30.421
12	HOXB7	28.461
13	HHEX	27.973
14	GATA2	23.984
15	HCLS1	23.087
16	HOXA5	20.052
17	TBX18	19.162
18	HOXB3	18.896
19	BNC1	12.005
20	ZEB1	11.675
21	KLF9	10.852
22	HOXD1	10.312
23	NFIB	10.1
24	HLX	9.854
25	NPAS2	9.726
26	FOSL2	9.72
27	ELF4	8.969
28	KLF2	8.559
29	FOXC1	7.728
30	HOXA11	7.292
31	HOXA10-HOXA9///HOXA9 ///MIR196B	7.169
32	ATF6	6.881
33	FOXF1	6.471
34	ZNF521	6.404
35	ZBTB38	5.751
36	IRF6	5.398
37	MMP14	5.361
38	ETS1	5.245
39	STAT6	5.227

**Table 4:** Genes in transcription factor category that are downregulated in endothelial cells.

	Gene	Fold Change
1	ZIC2	−506.971
2	SOX2	−243.085
3	ZIC5	−170.82
4	ZIC3	−143.398
5	OTX2	−120.52
6	SOX11	−79.857
7	SALL1	−46.361
8	SOX21	−46.13
9	SALL2	−42.04
10	ZNF90	−38.389
11	ZNF423	−36.361
12	SOX9	−27.58
13	BCL11A	−23.86
14	MYCN	−23.094
15	TFAP2C	−20.154
16	PBX1	−16.377
17	MKX	−13.612
18	ZNF154	−13.29
19	HESX1	−12.089
20	EBF1	−11.098
21	CUX2	−10.449
22	ESRRG	−10.06
23	HES6	−9.416
24	E2F5	−9.012
25	MYB	−8.481
26	POU3F1	−8.471
27	ZNF649	−8.279
28	SCAND3	−7.674
29	TRERF1	−7.328
30	DLX1	−7.172
31	ZNF93	−7.085
32	TAF4B	−6.925
33	ZNF398	−6.401
34	CITED1	−5.991
35	ZFP37	−5.643

**Table 5:** Genes in transcription factor category that are downregulated in endothelial cells and upregulated in neural cells.

	Gene	EC-Fold Change	Nn-Fold Change
1	IRX3	−21.279	6.33
2	IRX2	−10.615	6.497

**Table 6:** Genes in transcription factor category that are upregulated in neural cells.

	Gene	Fold Change
1	TFAP2B	113.974
2	TFAP2A	62.803
3	PAX6	51.035
4	POU3F2	24.153
5	NR2E1	22.86
6	ST18	19.396
7	ASCL1	17.971
8	PITX2	17.764
9	HOXA1	16.684
10	ARX	14.939
11	MAF	13.489
12	NR2F1	13.313
13	FOXG1	12.871
14	MEIS1	11.929
15	DLX5	11.139
16	ZEB2	11.09
17	TWIST1	9.671
18	IRX5	9.371
19	DMRT2	9.171
20	WNT5A	8.17
21	POU4F1	7.833
22	NEUROD1	7.794
23	MSX2	7.759
24	DLX6	7.711
25	SIX3	7.682
26	MEIS2	7.505
27	ONECUT2	6.997
28	ZFHX4	6.941
29	HOXC6	6.668
30	MAFB	6.001
31	TSHZ2	5.903
32	CBFA2T3	5.476
33	HOXC4	5.247
34	CSRNP3	4.986
35	ISL1	4.49
36	HOXC8	4.382
37	ZHX1	3.944
38	EMX2	3.913
39	ALX1	3.862
40	RUNX1T1	3.694
41	NEUROG1	3.44
42	SNAI2	3.352
43	LHX2	3.162
44	GRHL3	3.14
45	HOXD3	3.139
46	OTP	3.021
47	LHX9	2.848

**Table 7:** Genes in the transcription factor category that are downregulated in neural cells.

	Gene	Fold Change
1	FOXO1	−6.445
2	NFE2L3	−4.486
3	ZSCAN10	−3.712
4	MEOX2	−3.541
5	HMX2	−3.477
6	TCF19	−3.131
7	ARNTL2	−3.127

**Table 8:** Genes in epigenetic category that are downregulated in both endothelial and neural cells.

	Gene	EC-Fold Change	Nn-Fold Change
1	DNMT3B	−44.269	−5.983
2	PRDM14	−14.327	−3.042
3	HELLS	−5.666	−9.624

**Table 9:** Genes in epigenetic category that are upregulated in endothelial cells.

	Gene	Fold Change
1	PCGF5	9.831
2	LOXL2	7.979
3	ERCC6	5.444

**Table 10:** Genes in epigenetic category that are downregulated in endothelial cells.

	Gene	Fold Change
1	LIN28A	−616.831
2	LIN28B	−254.621
3	TET1	−62.007
4	TRIM71	−51.797
5	JARID2	−12.625
6	PRKCB	−8.155
7	CECR2	−6.632
8	H2AFY2	−5.827

**Table 11:** Genes in epigenetic category that are upregulated in neural cells.

	Gene	Fold Change
1	BCORL1	3.425
2	LHX2	3.162

**Table 12:** Genes in epigenetic category that are downregulated in neural cells.

	Gene	Fold Change
1	HENMT1	−2.912

## Data Availability

The data supporting this study are available upon request from the corresponding author NJ.
